# CovidCTNet: an open-source deep learning approach to diagnose covid-19 using small cohort of CT images

**DOI:** 10.1038/s41746-021-00399-3

**Published:** 2021-02-18

**Authors:** Tahereh Javaheri, Morteza Homayounfar, Zohreh Amoozgar, Reza Reiazi, Fatemeh Homayounieh, Engy Abbas, Azadeh Laali, Amir Reza Radmard, Mohammad Hadi Gharib, Seyed Ali Javad Mousavi, Omid Ghaemi, Rosa Babaei, Hadi Karimi Mobin, Mehdi Hosseinzadeh, Rana Jahanban-Esfahlan, Khaled Seidi, Mannudeep K. Kalra, Guanglan Zhang, L. T. Chitkushev, Benjamin Haibe-Kains, Reza Malekzadeh, Reza Rawassizadeh

**Affiliations:** 1 Health Informatics Lab, Metropolitan College, Boston University, Boston, USA; 2 Department of Biomedical Engineering, Amirkabir University of Technology, Tehran, Iran; 3 Department of Radiation Oncology, Massachusetts General Hospital, Harvard Medical School, Boston, USA; 4 Princess Margaret Cancer Centre, University of Toronto, Toronto, Canada; 5 Department of Medical Biophysics, University of Toronto, Toronto, Canada; 6 Department of Medical Physics, School of Medicine, Iran university of Medical Sciences, Tehran, Iran; 7 Department of Radiology, Massachusetts General Hospital, Harvard Medical School, Boston, USA; 8 Joint Department of Medical Imaging, University of Toronto, Toronto, Canada; 9 Department of Infectious Diseases, Firoozgar Hospital, Iran University of Medical Sciences, Tehran, Iran; 10 Department of Radiology, Shariati Hospital, Tehran University of Medical Sciences, Tehran, Iran; 11 Department of Radiology and Golestan Rheumatology Research Center, Golestan University of Medical Sciences, Gorgan, Iran; 12 Department of Internal Medicine, Iran University of Medical Sciences, Tehran, Iran; 13 Department of Radiology, Iran University of Medical Sciences, Tehran, Iran; 14 Institute of Research and Development, Duy Tan University, Da Nang, Vietnam; 15 Health Management and Economics Research Center, Iran University of Medical Sciences, Tehran, Iran; 16 Department of Medical Biotechnology, School of Advanced Medical Sciences, Tabriz University of Medical Sciences, Tabriz, Iran; 17 Department of Computer Science, Metropolitan College, Boston University, Boston, USA; 18 Department of Computer Science, University of Toronto, Toronto, ON Canada; 19 Ontario Institute for Cancer Research, Toronto, ON Canada; 20 Vector Institute for Artificial Intelligence, Toronto, ON Canada; 21 Digestive Disease Research Center, Tehran University of Medical Sciences, Tehran, Iran

**Keywords:** Biological techniques, Image processing

## Abstract

Coronavirus disease 2019 (Covid-19) is highly contagious with limited treatment options. Early and accurate diagnosis of Covid-19 is crucial in reducing the spread of the disease and its accompanied mortality. Currently, detection by reverse transcriptase-polymerase chain reaction (RT-PCR) is the gold standard of outpatient and inpatient detection of Covid-19. RT-PCR is a rapid method; however, its accuracy in detection is only ~70–75%. Another approved strategy is computed tomography (CT) imaging. CT imaging has a much higher sensitivity of ~80–98%, but similar accuracy of 70%. To enhance the accuracy of CT imaging detection, we developed an open-source framework, CovidCTNet, composed of a set of deep learning algorithms that accurately differentiates Covid-19 from community-acquired pneumonia (CAP) and other lung diseases. CovidCTNet increases the accuracy of CT imaging detection to 95% compared to radiologists (70%). CovidCTNet is designed to work with heterogeneous and small sample sizes independent of the CT imaging hardware. To facilitate the detection of Covid-19 globally and assist radiologists and physicians in the screening process, we are releasing all algorithms and model parameter details as open-source. Open-source sharing of CovidCTNet enables developers to rapidly improve and optimize services while preserving user privacy and data ownership.

## Introduction

In the era of communication, the current epidemic of highly contagious Covid-19 (SARS-Cov-2) has negatively impacted the global health, trade, and economy. To date, the mortality rate of Covid-19 is estimated to be 35–45 times higher than the pandemic influenza, accounting for more than 1,000,000 deaths^1–4^. Covid-19 has surpassed its predecessors SARS-CoV, and MERS-CoV, in morbidity and mortality^5^. Unfortunately, the long-term studies on SARS-CoV, the cause of SARS^6^, did not find effective and safe treatments^7^. Lack of effective therapy underlines the importance of early diagnosis, rapid isolation, and strict infection control to minimize the spread of Covid-19.

Currently, diagnosis is mainly based on the patient’s medical history, RT-PCR, and CT imaging^8–12^. High error (30–35%) of RT-PCR^8,9,13^, lack of distinction between viral contamination versus disease-bearing individuals^14^ or false-positive/negative^15^ may have contributed to the high prevalence of Covid-19 and the dismal therapeutic outcomes. Here, CT imaging plays a critical role in Covid-19 diagnosis since it not only detects the presence of disease in the lung but also enables identifying the stage of the disease by scoring the CT images^9,16,17^. CT imaging, however, has its own limitations that need to be addressed. The lack of specificity and the similarities between the lung lesions generated by other types of viral infection or community-acquired pneumonia (CAP) may contribute to misdiagnosis for Covid-19^18–20^. We hypothesized that using robust tools such as machine learning can resolve the CT imaging technical bias and corrects for human errors^17,21–26^.

An appropriate machine learning framework for Covid-19 detection should (i) be able to assist radiologists and their staff to rapidly and accurately detect Covid-19, (ii) be compatible with a wide range of image scanning hardware’s, and (iii) be user friendly to the medical community without computer-science expertize. In our effort to address the clinical diagnostic needs in the Covid-19 pandemic crisis under institutional review board (IRB) approval (IR.TUMS.VCR.REC.1399.007), we designed CovidCTNet framework. CovidCTNet is composed of a pipeline of deep learning algorithms trained on identifying Covid-19 lesions in lung CT images to improve the process of Covid-19 detection.

While deep learning approaches used for Covid-19 detection require large datasets^27–29^, CovidCTNet by employing BCDU-Net^30^ requires only a small sample size for training to achieve accurate detection of Covid-19 without potential bias. For these reasons, our model is significantly different from other models^25,29,31,32^ which requires a large dataset of CT images. In our framework, we first applied multiple pre-processing steps on CT images using BCDU-Net^30^ which is designed based on the U-Net^33,34^, a well-known convolutional network for biomedical image analysis. BCDU-Net is an optimum network due to memory (LSTM cells), allowing the model to remember the structure of the healthy lung. In particular, the CovidCTNet used BCDU-Net to (i) clean images, i.e., removing the image segments unrelated to infection, such as heart, skin, or the bed of CT image device and (ii) train a noise cancellation model, which was used by our model to extract infection. Note that both Covid-19 and CAP are associated with a lung infection, and visually they are very similar. Therefore, a robust Covid-19 identification approach should distinguish them accurately. Otherwise, classification algorithms cannot distinguish Covid-19, CAP, and control lungs in the small dataset and from original CT images. However, with the assistance of BCDU-Net, our model cleaned the CT images from other tissues, except the lung infection.

After the process of infection extraction, the result of CT images was fed into a convolutional neural network (CNN) to classify the given CT images as control, CAP, or Covid-19. All of our codes, including details of model parameters, are clearly explained and released as open-source. In this study, we developed the CovidCTNet, which consists of a pipeline of deep learning algorithms to accurately detect Covid-19 infection. A heterogeneous dataset was analyzed in this study to ensure that CovidCTNet can address the needs of hospitals across the globe, irrespective of the sample size, an imaging device (hardware), or the imaging software.

## Results

### Extraction of Covid-19 lesions from CT images

We assessed a dataset consisting of 16,750 slices of all CT scan images from 335 patients. Among this dataset, 111 (5550 CT slices) patients were infected with Covid-19 with a confirmed RT-PCR, patient’s medical history, and radiologist diagnosis. The second cohort was 115 (5750 CT slices) patients infected with CAP or other viral sources with CT images that can be potentially misdiagnosed for Covid-19. Our Control group consists of a cohort of patients 109 (5450 CT slices) with healthy lungs or other non-Covid-19/non-CAP diseases. Additionally, a cohort of 70 CT scans was used from SPIE-AAPM-NCI lung nodule classification challenge dataset^35^, a heterogeneous dataset that contains lung cancer as well (summarized in Tables 1 and 2). 66 cases (21,888 CT slices) out of 70 were randomly selected for training and validation phases. Four cases served as a control for reader tests. CT images were acquired from multiple institutions, including five medical centers in Iran, a country that is highly affiliated with Covid-19 and from publicly available dataset from lung nodule classification (LUNGx) challenge, an archive generated by the University of Chicago^35,36^.

**Table 1:** Detail information of the samples in multiple steps of analysis (preprocessing, train, validation, and test phases).

#Patient cohort	Control		Total		Loss (binary cross-entropy) Optimizer = Adam learning rate = 0.001
	CT slices with Perlin noise	CT slices without Perlin noise	Patients	CT slices	
Preprocessing-train	9913	9914	60	19,827	0.3585
Preprocessing-validation	1031	1030	6	2061	0.3638

Details of individual samples and total cases that were used in preprocessing.

**Table 2:** Detail information of the samples in multiple steps of analysis (preprocessing, train, validation, and test phases).

#Patient cohort	Control		CAP		Covid-19		Total	
	Patients	CT slices	Patients	CT slices	Patients	CT slices	Patients	CT slices
Train	100	100 × 50	100	100 × 50	100	100 × 50	300	15,000
Validation	5	5 × 50	5	5 × 50	5	5 × 50	15	750
Reader test	4	4 × 50	10	10 × 50	6	6 × 50	20	1000
Total	109	109 × 50	115	115 × 50	111	111 × 50	335	16,750

A summary of individual samples and total cases that were used in the train, validation, and test phases. To maintain the balance in the dataset, a total number of 100 cases were used for each of Control, CAP, and Covid-19 groups.

The dataset was collected from 12 different CT scanner models of five different brands. Our sample size was small and to achieve a high performing model that is operational and unbiased, we used BCDU-Net as the backbone of our model. To identify Covid-19 in the lung as well as CAP lesion, we generated pseudo-infection anomalies in the CT control images using Perlin noise^37^.

### CovidCTNet mitigates the challenge of small dataset by highlighting the infection

**Fig. 1:**
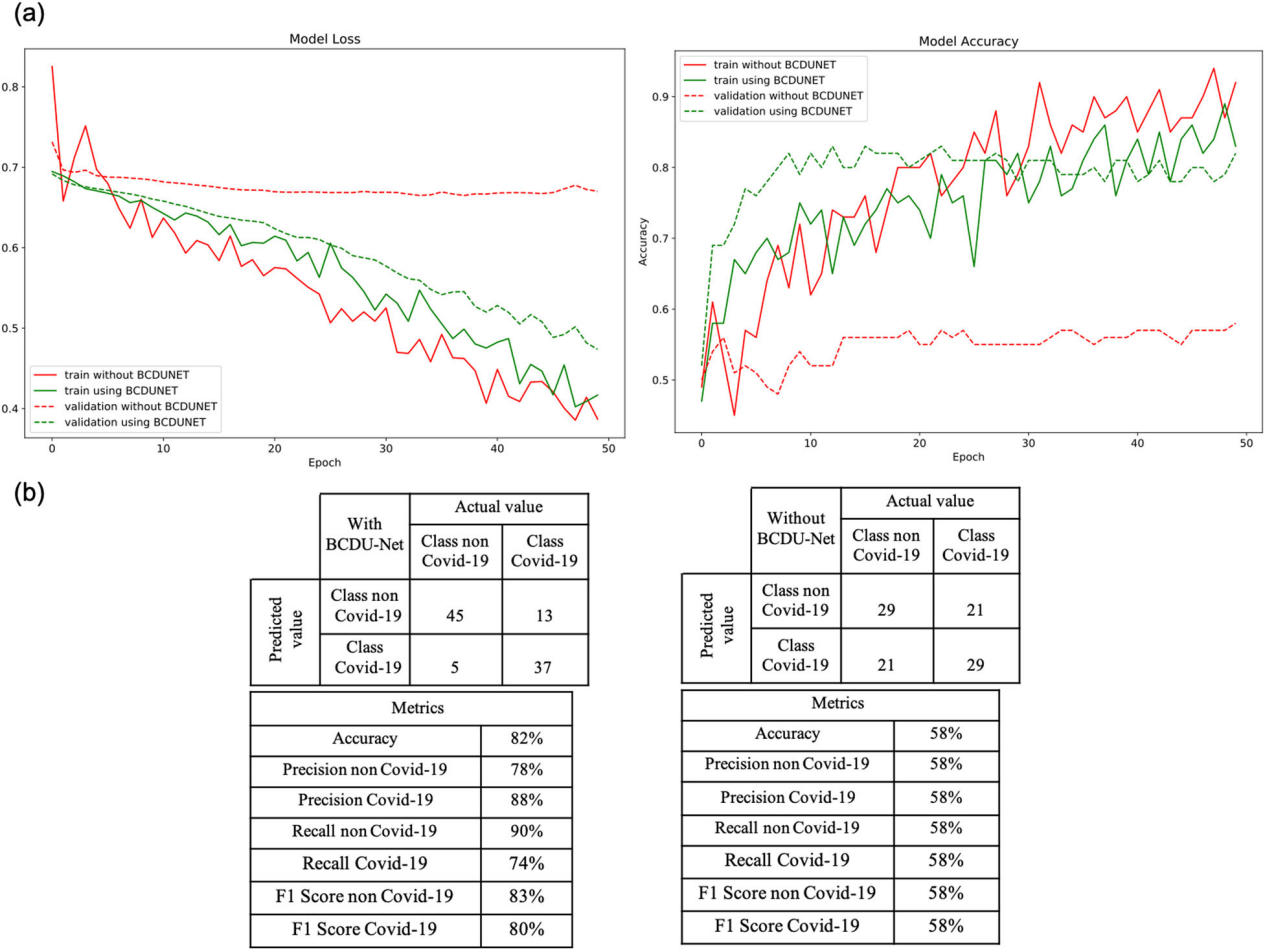
BCDU-Net increases the robustness of the CNN model. **a** To show the effect of BCDU-Net on the preprocessing, the procedure was done with and without applying BCDU-Net/Perlin noise. The outcome of the model is presented with respect to loss and accuracy. **b** The confusion matrix and other classification related metrics in detail. The results shown in this figure are based on just 50 randomly selected cases for each class of Covid versus non-Covid.

**Fig. 2:**
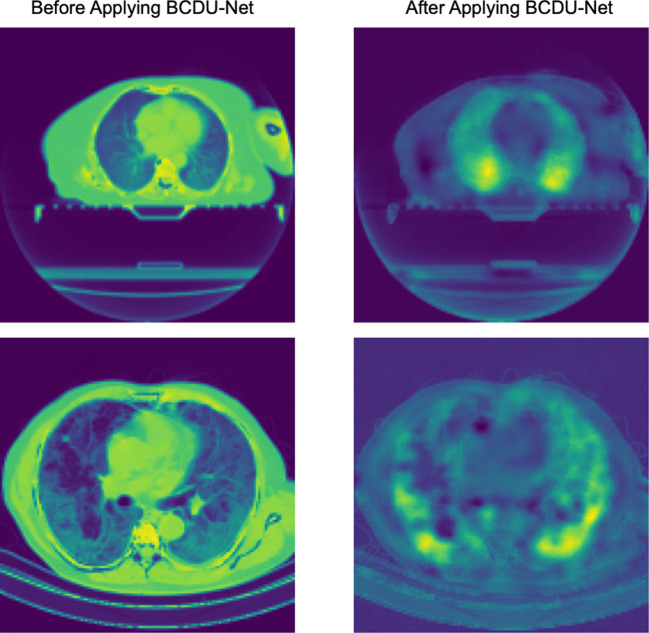
Covid-19 and CAP infection extraction by BCDU-Net. The filtered images (left) will be used for classification by CNN. An unprocessed 3D image of the whole lung infected with Covid-19 is shown in Fig. 3a. The same image was processed with BCDU-Net to remove non lung-related parts and to extract and highlight the Covid-19 infection (Fig. 3b).

**Fig. 3:**
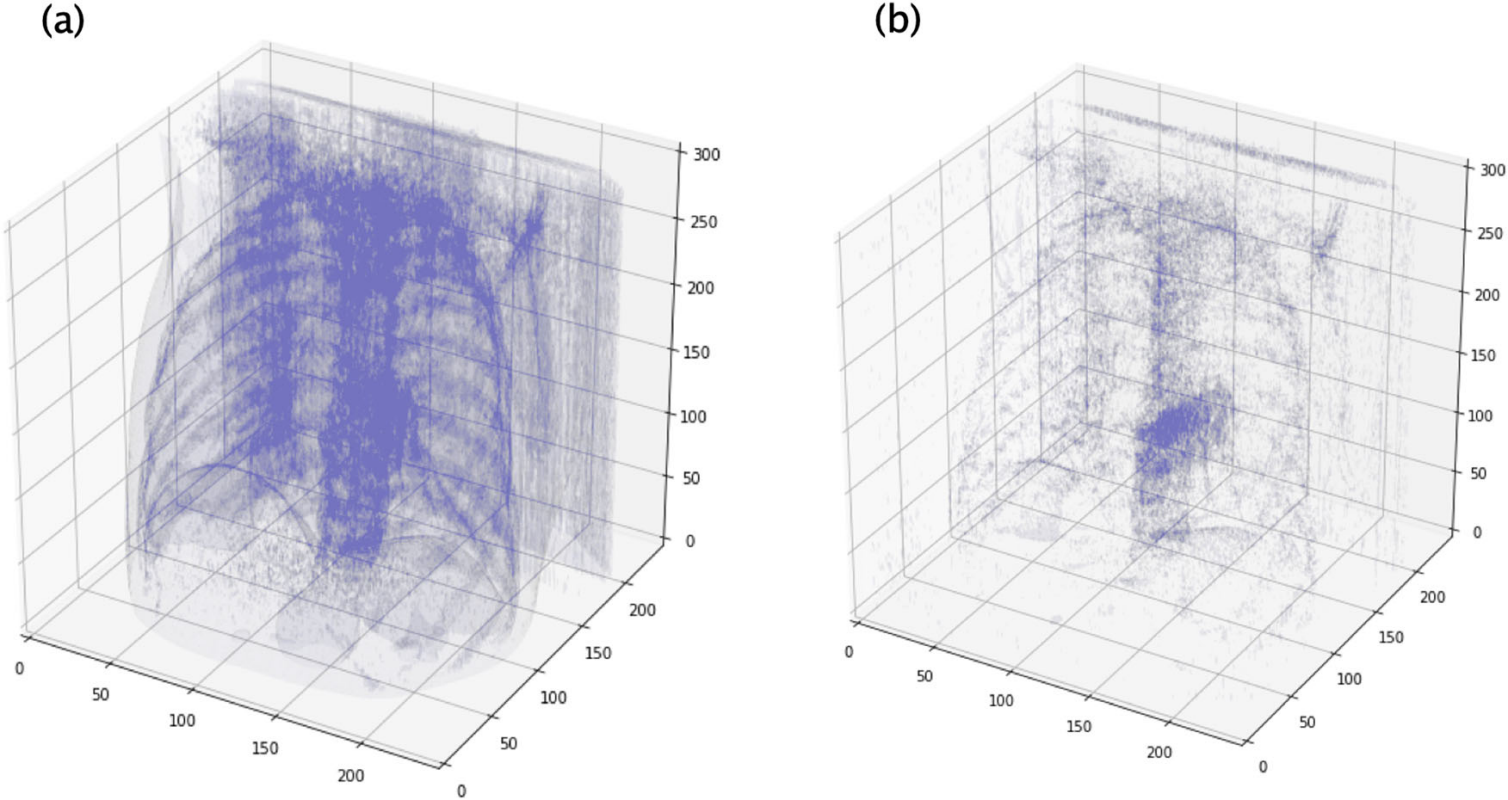
Schematic representation of BCDU-Net module to detect the infection in CT images. **a** The original CT images visualized in point cloud. **b** Reconstructed lung image acquired by feeding the CT slices (Fig. 8 middle part h) into BCDU-Net. The Covid-19 infection area is highlighted in **b**.

To test whether applying Perlin noise and using BCDU-Net is necessary for preprocessing and if they increase the accuracy of our model, we conducted a validation experiment. The 3D CNN model was performed with and without the use of BCDU-Net and Perlin noise. The implementation of BCDU-Net significantly boosted the accuracy of the model and demonstrated the importance of using Perlin noise and preprocessing steps. Figure 1 presents binary cross entropy (loss) and accuracy of the CovidCTNet in different conditions. While the accuracy of the model without using BCDU-Net and Perlin noise at the training phase is very high, it drops significantly in the validation phase. This confirms that the features and parameters that were selected by the CNN model (without BCDU-Net) were not sufficient. In addition, applying them significantly changes the accuracy of training and validation, which demonstrates the need for preprocessing in increasing the model robustness. Note that the results shown in Fig. 1a, b were generated by training the model with only 50 cases for each class, which proves the necessity and usefulness of applying the BCDU-Net for a limited amount of data. Figure 2 presents the extracted infection by BCDU-Net in 2D and Fig. 3 presents the extracted infection by BCDU-Net in 3D. The output of BCDU-Net (Fig. 3) will be fed to the CNN model as an input. It can be seen from Figs. 2 and 3 how BCDU-Net reduces the noninfectious parts of the CT image and highlights the infections inside the lung.

### CovidCTNet accurately detects Covid-19 from other lung diseases

**Fig. 4:**
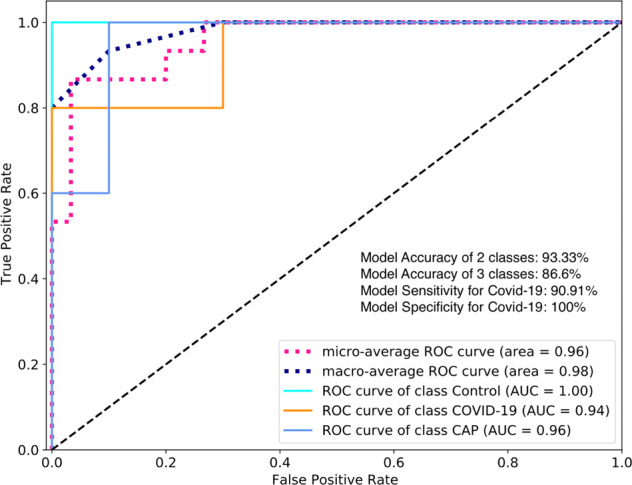
Performance of CovidCTNet in detecting Control, Covid-19, and CAP. The model’s AUC for Covid-19 detection is 0.94 (*n* = 15 cases). The accuracy, sensitivity, and specificity of the model are shown. The model operation in three classes demonstrates the detection of all three classes including Covid-19 versus CAP and versus Control and in two classes indicates the detection of Covid-19 as one class versus non-Covid-19 (CAP and Control) as second class.

The output of the algorithm (a tensor such as the right side of Fig. 3b) was fed into the CNN classification algorithm. In CNN assessment, the dataset was split in 95% to train the algorithm, and 5% to validate the model in the hold-out. The area under receiver operating characteristics (ROC) curve (AUC) for Covid-19 at the validation phase was 94%, with an accuracy of 93.33% when CNN classified Covid-19 versus non-Covid-19 (two classes) (Fig. 4). CNN achieved the accuracy of 86.66% when it classified Covid-19 versus CAP and Control (three classes). The detection sensitivity of 90.91% and specificity of 100% were recorded for Covid-19 (Fig. 4).

### CovidCTNet outperforms radiologists

To test the classification quality of our framework, an independent dataset consisting of 20 cases mixed of Control, Covid-19, and CAP were assessed using our framework and in parallel four certified and independent radiologists who were not involved in the process of data collection. The average reader performance of four radiologists showed a sensitivity of 79% for Covid-19 and specificity of 82.14%. The CNN classification of CovidCTNet, however outperformed the radiologists and achieved Covid-19 detection with sensitivity and specificity of 93 and 100%, respectively. Table 3. details the comparison of radiologist performance versus CovidCTNet.

**Table 3:** Comparison of the accuracy of CovidCTNet versus radiologists.

	Precision	Recall	*F*1-score
*Radiologist 1*			
Control	1	0.5	0.533
CAP	0.8	0.4	0.533
Covid-19	0.385	0.833	0.526
Accuracy	0.55		
*Radiologist 2*			
Control	0.5	0.5	0.5
CAP	0.889	0.8	0.842
Covid-19	0.714	0.833	0.769
Accuracy	0.75		
*Radiologist 3*			
Control	1	1	1
CAP	1	1	1
Covid-19	1	1	1
Accuracy	1		
*Radiologist 4*			
Control	0.25	0.5	0.33
CAP	0.66	0.6	0.63
Covid-19	1	0.5	0.66
Accuracy	0.55		
*All radiologist average*			
Control	0.68	0.625	0.624
CAP	0.837	0.7	0.751
Covid-19	0.774	0.791	0.738
Accuracy	0.71		
*CovidCTNet*			
Control	0.6	0.75	0.67
CAP	0.9	0.9	0.9
Covid-19	1	0.83	0.91
Accuracy	0.85		

Table 2 summarizes the precision (positive predictive value), recall (sensitivity), accuracy, and *F*-score of each radiologist in comparison to the CovidCTNet.

**Fig. 5:**
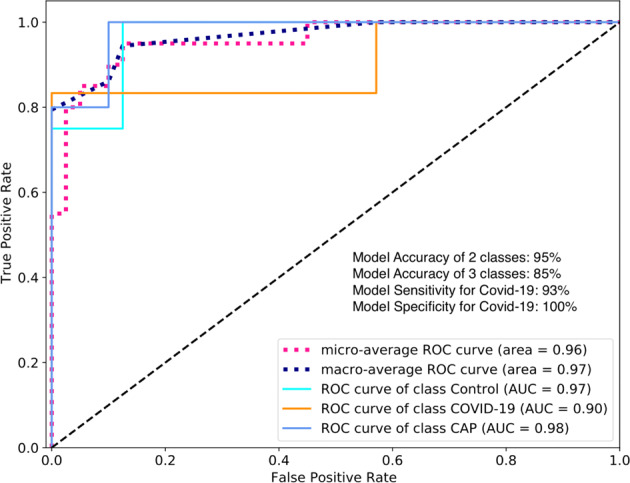
Comparison of the outcome of CovidCTNet versus reader study. Performance of model and radiologists (reader) in a pool of chest CT dataset mixed of control, Covid-19 and CAP. AUC of Covid-19 is 0.90 (*n* = 20 cases). The accuracy, sensitivity, and specificity of readers versus model are shown. The model operation in three classes demonstrates the detection of all three classes including Covid-19, CAP, and control separately and in two classes indicates the detection of Covid-19 as one class versus CAP and control as second class. While macroaverage takes the metric of each class independently and computes their average, the microaverage computes the average metric after aggregating the contributions of all classes.

Radiologists performance accuracy was 81%, while CovidCTNet classification achieved a 95% accuracy when the question was detecting between Covid-19 versus non-Covid-19 (2 classes). When we asked to detect Covid-19 versus CAP versus control (three classes), again our approach outperformed the radiologists with an accuracy of 85% compared with human accuracy of 71%. The AUC of the model in Covid-19 detection versus reader test was 90% (Fig. 5). The accuracy, sensitivity, and specificity of the model showed a significantly higher validity compared to the average of radiologists.

### Images of Covid-19 and CAP infection share structural similarities

**Fig. 6:**
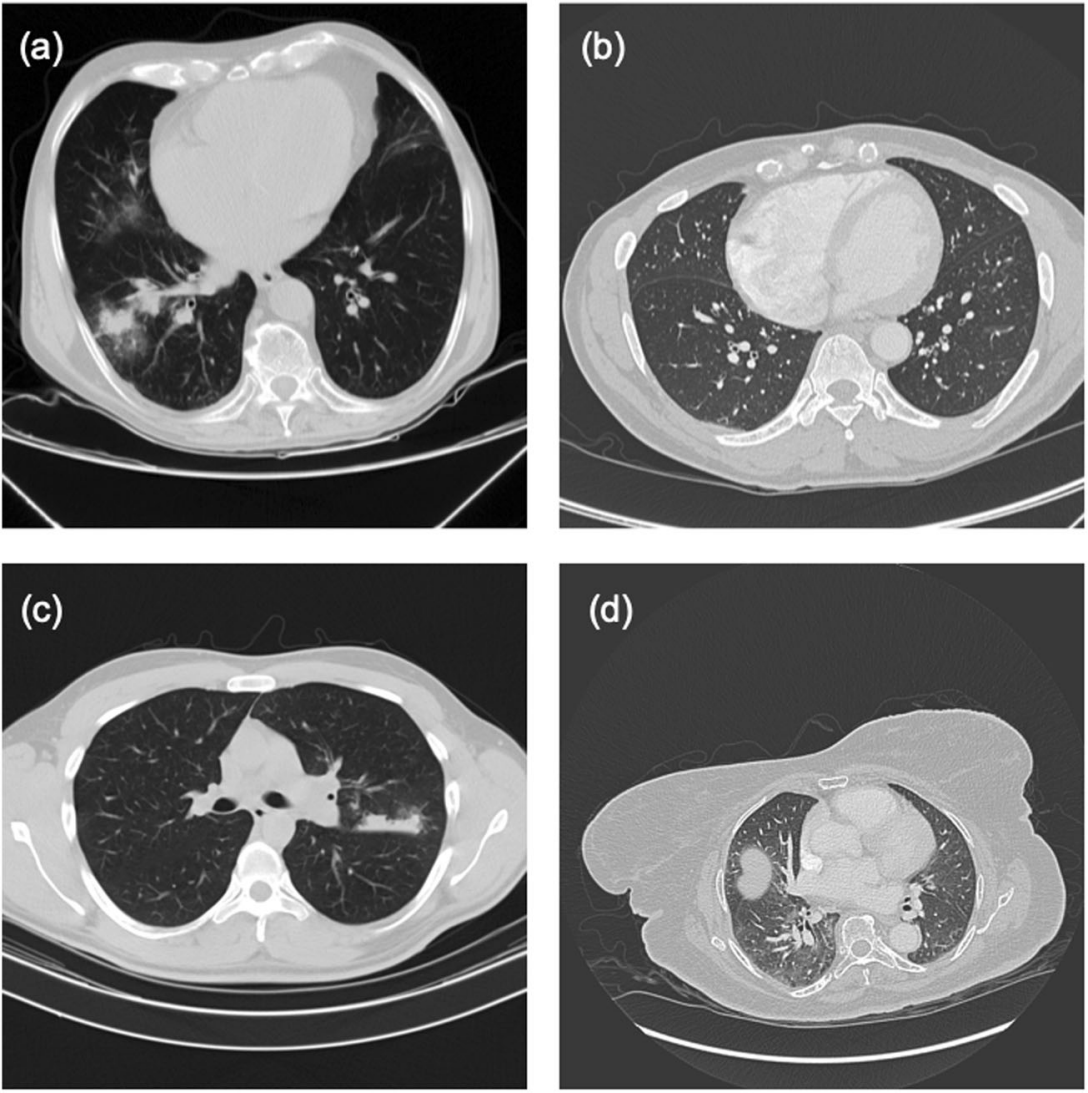
Representative examples of CT images used to test the performance of CovidCTNet versus radiologists. **a** A CT image of CAP. This image is misidentified as Covid-19 or control by two out of four radiologists and correctly diagnosed by CovidCTNet. **b** A CT image of control, that was misdiagnosed by three out of four radiologists as Covid-19 or CAP and correctly diagnosed by CovidCTNet as control. **c** A sample of Covid-19 that was detected as Control by CovidCTNet and as Control or CAP by three out of the entire panel of radiologists (four members). **d** Image of control that was misdiagnosed by the CovidCTNet as CAP and by two radiologists as Covid-19 or control. Note that, in this figure one single slide of the entire scan is shown as a representative of all CT images of a patient.

Despite some clear differences in Covid-19 and CAP infection pattern, the high similarities in ground-glass opacities (GGO) and consolidation on chest CT of Covid-19 and CAP (Fig. 4) makes differential detection a challenge. Several suspicious and challenging images are shown in Fig. 6.

The CAP CT slide (Fig. 6a) was misdiagnosed by radiologists as Covid-19 or Control by radiologists but correctly by CovidCTNet. Figure 6b is a control image that was correctly diagnosed by CovidCTNet and misdiagnosed by three radiologists. Figure 6c is a Covid-19. Its diagnosis posed a challenge for three radiologists out of four and also for CovidCTNet. The Control shown in Fig. 6d was misdiagnosed by two radiologists and by CovidCTNet.

## Discussion

In recent studies the average sensitivity of radiologists to detect the Covid-19 infection is reported to be approximately 70%^20^, indicating the need for decision support tools to assist radiologists in detecting Covid-19, especially in regions where there is limited in the number of trained clinical staff or the comprehensive expertize to detect Covid-19.

Due to the high value of patch-based classification^38^ we implemented it as one of our models, which did not perform as good as CovidCTNet on the small dataset. The CovidCTNet improved the accuracy and consistency of lung screening for Covid-19 detection through a ready-to-use platform with a sensitivity of 93% and accuracy of 95% (in making a binary decision, i.e., Covid-19 and non-Covid19). While the broad similarity of patterns and image features of Covid-19 and CAP posed a challenge for algorithm training, the high accuracy of the model indicates the potential for CovidCTNet to be further refined and adapted as a clinical decision support tool. In contrast to state-of-the-art works^17,25,26,29,31,32^ the dataset we used in this study is significantly smaller and highly heterogeneous. Adding more CT images will increase the accuracy and performance of the model.

Beyond optimizing and improving the Covid-19 detection, CovidCTNet has the potential to significantly impact the clinical workflow and patient care by offering a rapid, inexpensive, and accurate methodology to empower healthcare workers during the pandemic. Our radiologists are highly experienced from prestigious institutions. In an underrepresented region, it is not easy to find an experienced radiologist and we believe these types of AI systems will be significantly helpful to save lives. Importantly, when an infection type is hard to be diagnosed by the human eye, and when a consensus among radiologists cannot be made, CovidCTNet can be operated as a reliable source of diagnosis. To our knowledge, despite other promising efforts^25,29,39^, (summarized in Table 4) CovidCTNet is an open-source framework that allows researchers and developers to adjust and build other applications based on it in a fraction of time. Besides, our approach follows the guideline proposed by Mongan et al.^40^ for developing an AI method for medical image analysis.

**Table 4:** Comparison of state-of-the-art Covid-19 classification approaches which used CT images.

Citation	Characteristics	Algorithm	Outcome	# of analyzed patients (#of images)
Javaheri et al.	U-net-based image preprocessing	BCDU-Net and Perlin noise exposure; CNN	AUC: 90% Sensitivity: 93% Specificity: 100%	335 (16,750)
Bai et al.^20^	HU-based lung segmentation	EfficientNet B4;CNN	AUC: 87% Sensitivity: 89% Specificity: 86%	1186 (132,583)
Mei et al.^39^	A joint model using Lung segmentation and clinical data	ResNet-18; CNN; SVM; Random Forest; MLP	AUC: 92% Sensitivity: 84.3% Specificity: 82.8%	905 (unknown)
Li et al.^25^	U-net-based lung segmentation	Resnet50; CNN	AUC: 96% Sensitivity: 90% Specificity: 96%	3322 (4356)
Zhang et al.^29^	A joint model using Lung segmentation and clinical data. U-net, DRUNET, FCN, SegNet and DeepLabv3-based lung segmentation	3D Resnet-18; CNN	AUC: 97% Sensitivity: 92% Specificity: 85%	4154 (617,775)

This table provides a summary on existing models including their method, achieved AUC, sensitivity, and specificity. We report here the list of approaches that rely on chest CT scans. The X-ray images were excluded from this list as they have been studied by Maguolo et al.^41^.

In future efforts, we intend to (i) increase other samples as the CT scans using in this study are mostly from Iranian patients, (ii) include other demographic details of patients including age, gender and medical history to develop a predictive model, (iii) testing the model with a larger number of CT scan databases to further validate and broaden the application of our strategy.

## Methods

### Pre-processing

In our effort to address the clinical diagnostic needs, with the written informed consent of patients, under institutional review board (IRB) approval (IR.TUMS.VCR.REC.1399.007, Tehran University of Medical Sciences), we collected CT images. The CT images covered a variety of image sizes, slice thicknesses, and different configurations of a range of CT scanning devices. Depending on the device and the radiologist decision, the number of scans (e.g., 60, 70, etc.), the image resolution (e.g., 512 × 512 pixels, 768 × 768 pixels, etc.), and pixel spaces in the CT images varied. Together these factors allowed us to generate a heterogeneous collection that accounts for differences in CT imaging that exist among the medical community. This broad heterogeneity within the image collection aimed to resolve the potential bias in image analysis towards a specific image quality or types of CT imaging device^40,41^.

In the first step of pre-processing, the CT slices were resampled along three axes (*z, y, x*) to account for the variety of voxel dimensions among the CT slices (voxel is a single pixel, but in three dimensions). We used the distances of 1 × 1 × 1 mm for all voxel dimensions. Our method unified CT scans into the same scale and created a resampled dataset from the original dataset, known as resampling to an isomorphic resolution^42^ (https://www.kaggle.com/kmader/finding-lungs-in-ct-data) (Fig. 8 upper part). In the second pre-processing step, pixel value of the resampled CT images (3D) was optimized to have a proper range of Hounsfield Units (HU). In our dataset, the least dense object such as air takes a value of −1000. Lung is an organ filled with air and thus acquires a HU value of −700 to −600. Other organs that may interfere with our analysis include water (HU of 0), fat (HU of −90 to −120) soft tissue (HU of 100–300), and bone (HU of 300–1900).

**Fig. 7:**
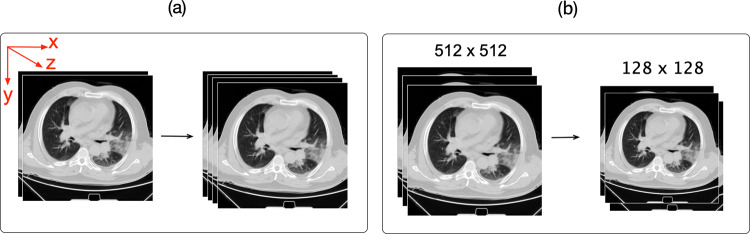
Schematic representation of the pre-processing phases. **a** Each patient’s CT image (3D) was resampled to isomorphic resolution, while *x* and *y* are the image coordinates and *z* represents the number of slices. **b** All CT slices (2D) with different sizes were resized to have 128 × 128 pixels on the *x* and *y* axis, but the *z* axis that depicts the number of slices remained intact. Here, a 512 × 512 pixels CT slice is resized into a 128 × 128 pixels CT slice.

Consequently, we filtered CT slices (2D) to remove non-lung tissue (e.g., skin, bone, or scanner bed) that may negatively impact our analysis and to keep only the lung related parts with an HU value ranging from −1000 to 400. Next, a min-max normalization is applied to rescale the −1000 and 400 numerical ranges of pixels to a 0.0 and 1.0 scale (Fig. 7b). In the third step of pre-processing, all CT slices of various pixel sizes were resized to a uniform 128 × 128 pixels on their *x* and *y* dimensions but the number of slices (*z*) remained intact (Fig. 7b).

### Algorithms

**Fig. 8:**
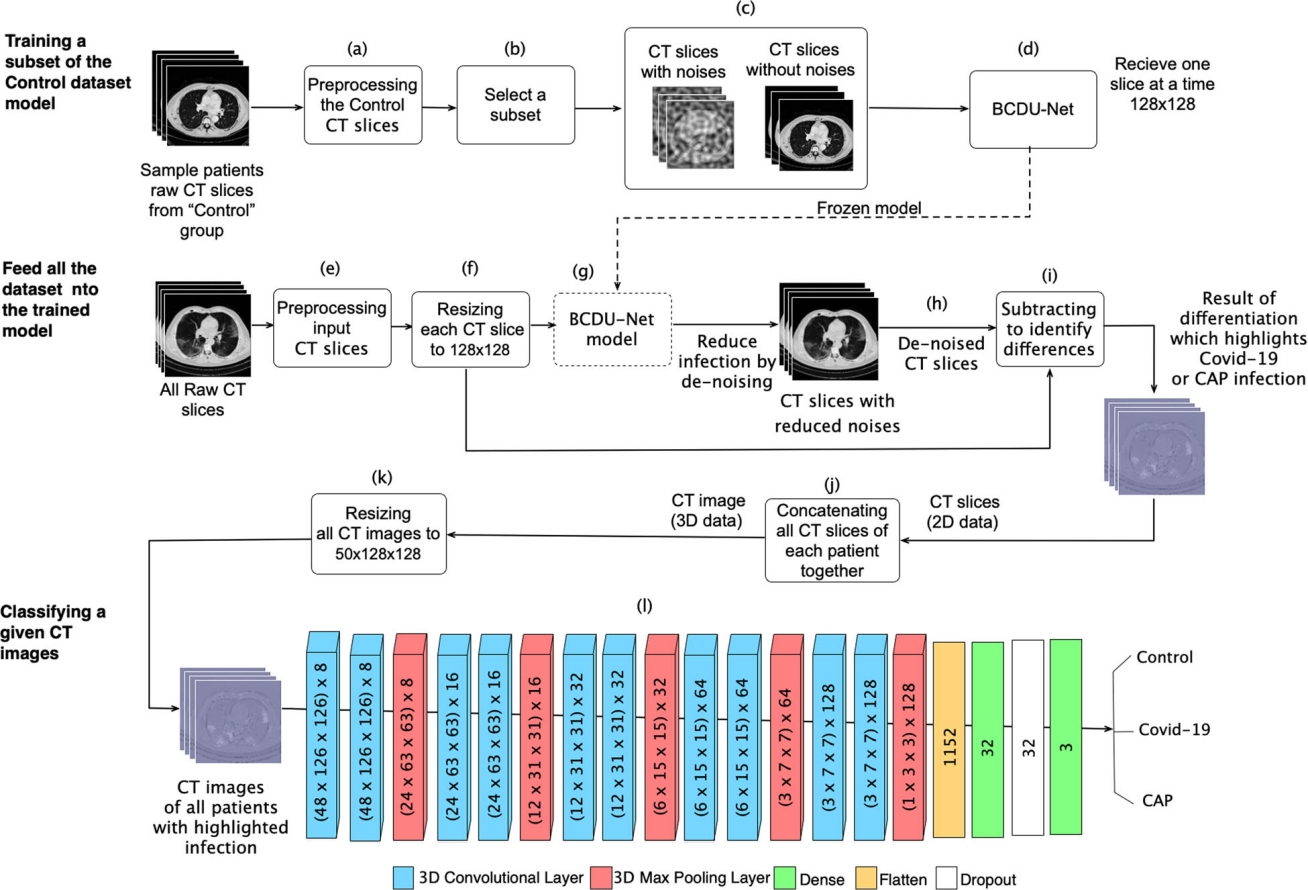
Multistep pipeline of deep learning algorithms to detect Covid-19 from CT images. Upper part, Training step of the model for learning the structure of Control CT slices. Middle part, Images subtracting and lung reconstructing from CT slices with highlighted Covid-19 or CAP infection (violet color). The results of step “i” are a 2D image. The slices at z axis concatenate to generate 3D CT image, the input of CNN model. Lower part, CNN model classifies the images that were constructed in the previous stage. To integrate this pipeline into an application the user needs to start from stage (middle part) and then the CNN algorithm recognizes whether the given CT images of a given patient presents Covid-19, CAP, or control. The number outside the parentheses in CNN model, present the number of channels in the CNN model.

The architecture of CovidCTNet is presented in detail in Fig. 8. To identify Covid-19 in the lung from CAP lesion, we generated pseudo-infection anomalies in the CT Control images using BCDU-Net^30^. The BCDU-Net module played a critical role in allowing the detection of infections that have numerous features in a small dataset. It helped us to increase the accuracy and the rate of model convergence by using the initialization of the model that is trained on the Kaggle dataset for lung segmentation (https://www.kaggle.com/kmader/finding-lungs-in-ct-data). The BCDU-Net was used in our model for two purposes, first cleaning images, by removing tissues that are not related to lung infection, such as heart, skin, or the bed of CT image device, and second canceling the noise, which is used by our model for lung infection identification. To cancel the noise, BCDU-Net focused on lung infection by generating Perlin noise^37^ (pseudo-infection) and detecting infections. A subset of Control images mixed of noisy and non-noisy were given to BCDU-Net as an input. At the same time, the original Control images of noisy or non-noisy subsets were targeted in the model as output, mimicking the Covid-19 and CAP anomalies in the Control cases (Fig. 8 upper part). The motivation of using artificial noise (to train the model for infection detection) was to simulate both the healthy and infected state of the same lung. Therefore, the BCDU-Net will learn the differences and how to extract infection from the CT images. To learn how to clean the CT images, BCDU-Net received original CT images without noise along with the images that have noise applied on them. Afterward, the model learnt to identify and tried removing unnecessary image contents such as heart tissue. By feeding the BCDU-Net with noisy CT images, the model learnt to identify infections or lesions.

To this end, we specified the input which is a combination of original healthy CT images and the CT images with noise. We defined the target of our model to be the same input CT images but without noise. Consequently, the output of BCDU-Net is de-noised and reconstructed CT images from the original and noisy CT images. Reconstructing the CT images helped the model to learn the pattern of the control lung and reconstruct the original Control image as output by noise reduction (de-noised) and infection removal (Fig. 8b, c). Thus, Covid-19 or CAP images could not be reconstructed correctly at this stage. Identifying the Control lung pattern led to recognition of non-control slices such as Covid-19 or CAP. In the first step, the training phase (Fig. 8 upper part) starts by randomly selecting a dataset of 66 control patients (21,888 slices) and applying the pre-processing steps on their CT images (Fig. 8a, b). The dataset was divided into two subsets: (i) the original CT images of 10,944 slices (Fig. 8c right), and (ii) CT images of 10,944 slices with applied Perlin noise^37^ (Fig. 8c left). The BCDU-Net model was trained with two noisy and non-noisy subsets of Control images and the trained model was frozen at this step (Fig. 8d).

Next, we applied pre-processing to the entire dataset. To ensure fair comparison, the images that were used in the pre-processing step (Fig. 8 upper part), were excluded from the validation step.

Afterwards these CT slices, including Control, CAP and Covid-19 were resized (Fig. 8 middle part e, f) and fed into the frozen BCDU-Net model (Fig. 8, middle part g). The output of the BCDU-Net is the de-noised CT slices (Fig. 8, middle part h). The algorithm subtracted the output of BCDU-Net, the lung slices without infection, (Fig. 8, middle part i) from the preprocessed CT slices, infected lung with Covid-19 or CAP (Fig. 8, middle part e, f) to acquire the infected areas of lung (Fig. 8, middle part i). Because the outcome of subtraction (Fig. 8, middle part i) depicted the highlighted infection area (Covid-19 and CAP) without other tissues or artifacts (Fig. 8, middle part image in violet color and Fig. 3b), it provided a reliable source for the infection classification as Covid-19 or CAP. Further examples of the result of this step are shown in Fig. 2, which shows exactly how Covid-19 and CAP infections were extracted by BCDU-Net. In other words, an example of the subtracted data (violet CT slices resulted from Fig. 8, middle part k) depicts the infection area in the lung (Fig. 3b).

In the validation, we observed that the subtraction resulted from original non-infected CT slices versus the output of BCDU-Net was insignificant, confirming the accuracy of detecting noise as an indication for the infected area. The slices at *z*-axis were concatenated to generate a 3D CT image that was the input of a three-dimensional convolutional neural network (CNN) model (Fig. 8, middle part j). The outcome of CT slices was resized due to high variation among the number of CT slices for each patient (Fig. 8, middle part k). Resizing ensures that all CT images have equal sizes, which is required by CNN to have a unified size (50 × 128 × 128). Here, 50 in the *z*-axis indicates that all the patients’ CT slices were resized to 50 slices. These 3D images were already labeled by radiologists as Covid-19, CAP, or control. To implement the classification algorithm, we used CNN. In the final step, the result of Fig. 8 middle part k was fed into the CNN model (Fig. 8, lower part l) as a training dataset. In the training phase, the model learned to distinguish Covid-19, CAP, and control. CNN model was then validated by using 15 cases that were selected randomly and were never used before in any of the training and preceding steps. The output of the CNN algorithm is a numerical value that classifies the given patient CT images as Covid-19 or CAP or control (Fig. 8, lower part l).

The slices at *z*-axis were concatenated to generate a 3D CT image that was the input of a three-dimensional convolutional neural network (CNN) model (Fig. 8, middle part j). The outcome of CT slices was resized due to high variation among the number of CT slices for each patient (Fig. 8, middle part k). Resizing ensures that all CT images have equal sizes, which is required by CNN to have a unified size (50 × 128 × 128). Here, 50 in the *z*-axis indicates that all the patients’ CT slices were resized to 50 slices. These 3D images were already labeled by radiologists as Covid-19, CAP, or control.

### Reporting summary

Further information on research design is available in the Nature Research Reporting Summary linked to this article.

## Supplementary information

Reporting Summary

## Data Availability

The raw image dataset generated or analyzed during this study is not publicly available due to the patient privacy/consent. Datasets are available to qualified researchers following completion of a Dataset License Agreement, which is available from the corresponding author’.

## Code availability

To allow full reproducibility of our claims^43,44^ all codes are available at https://github.com/mohofar/covidctnet. The repository contains all necessary information of model instruction and code execution. For model validation, some CT images of all three groups (Covid-19, CAP, and control) are included in the dataset.
